# Extreme spikes in DMS flux double estimates of biogenic sulfur export from the Antarctic coastal zone to the atmosphere

**DOI:** 10.1038/s41598-019-38714-4

**Published:** 2019-02-19

**Authors:** A. L. Webb, M. A. van Leeuwe, D. den Os, M. P. Meredith, H. J. Venables, J. Stefels

**Affiliations:** 1 School of Life Sciences, University of Warwick, Coventry, CV4 7AL UK; 2 GELIFES, University of Groningen, 9700 CC Groningen, The Netherlands; 3 Institute for Life Science and Technology, Hanze University of Applied Sciences, 9700 RM Groningen, The Netherlands; 4 British Antarctic Survey, High Cross, Cambridge, CB3 0ET UK

## Abstract

Biogenic dimethylsulfide (DMS) is a significant contributor to sulfur flux from the oceans to the atmosphere, and the most significant source of aerosol non sea-salt sulfate (NSS-SO_4_^2−^), a key regulator of global climate. Here we present the longest running time-series of DMS-water (DMS_W_) concentrations in the world, obtained at the Rothera Time-Series (RaTS) station in Ryder Bay, West Antarctic Peninsula (WAP). We demonstrate the first ever evaluation of interseasonal and interannual variability in DMS_W_ and associated flux to the atmosphere from the Antarctic coastal zone and determine the scale and importance of the region as a significant source of DMS. Impacts of climate modes such as El Niňo/Southern Oscillation are evaluated. Maximum DMS_W_ concentrations occurred annually in January and were primarily associated with sea-ice break-up. These concentrations resulted in extremely high (up to 968 µmol m^−2^ d^−1^) DMS flux over short timescales, which are not parameterised in global-scale DMS climatologies. Calculated DMS flux stayed above the aerosol nucleation threshold of 2.5 µmol m^−2^ d^−1^ for 60% of the year. Overall, using flux determinations from this study, the total flux of DMS-sulfur from the Austral Polar Province (APLR) was 1.1 Tg sulfur yr^−1^, more than double the figure suggested by the most recent DMS climatologies.

## Introduction

Dimethylsulfide (DMS) is a semi-volatile organic sulfur compound produced in surface waters around the globe, primarily from the breakdown of the algal osmolyte dimethylsulfoniopropionate (DMSP)^1,2^. Approximately 10% of total global DMS production ventilates through the sea-air interface^3,4^, where it accounts for approximately 50% of the natural global atmospheric sulfate burden^5^. Current estimates calculate a global DMS flux to the atmosphere of 28.1 (17.6–34.4) Tg S per year^6^, which is approximately half the anthropogenic global atmospheric sulfur input^6,7^. This makes DMS an important contributor to global sulfur fluxes. Once in the atmosphere, DMS oxidation contributes to the formation of non sea-salt sulfate (NSS-SO_4_^2−^), a cloud condensation nuclei (CCN) important for cloud formation and thereby reducing incoming solar radiation, and subsequently cooling the climate^8^. Charlson and co-authors postulated a negative feedback loop between global temperature and DMS production that would keep Earth’s temperature in homeostasis; this hypothesis is referred to as the CLAW-hypothesis. The hypothesis has recently been questioned^9^ and model calculations on the future response of DMS to changes in global temperature vary widely: both increases^10,11^ and decreases^12^ in surface water DMS concentrations are predicted, with corresponding variation in Southern Ocean DMS flux between the models^6,13^. These results indicate large uncertainties in the processes surrounding DMS production, and emphasise the need to generate improved mechanistic understanding so to amend model parameterisation of the DMS flux.

Global climatologies of DMS concentrations show that the polar regions are of significant importance to total global DMS production, in particular the Southern Ocean^6,13^. The total Southern Ocean (south of 40°S) DMS flux is calculated at approximately 3.4 Tg S during summer months (December to February)^14^, with an annual total flux estimated at 5.8 Tg S^6,15^. However, calculations of the DMS climatologies are still highly uncertain due to two factors. Firstly, the highest DMS concentrations (above 148 nmol L^−1^), as found in the marginal ice zone, are omitted because data are highly variable and scarce^6^. Secondly, availability of austral winter data is extremely limited^6,14^. A recent update of the summertime climatology, however, indicated that extremely high DMS concentrations might be a real feature in some areas of the Southern Ocean^14^; in the short, highly productive Antarctic spring and summer seasons, surface water DMS concentrations can exceed 50 nmol L^−1 ^^16–18^. These high values are often associated with the release of ice algae, recognised as a significant source of DMSP^19–21^, from melted sea ice^21–24^. DMSP is an important osmolyte and cryoprotectant and may play a pivotal role in sea-ice algae surviving the extreme conditions of temperature and salinity that prevail in sea ice^25^. The release of DMSP during ice melt events may be the source of significant input of DMS into polar waters, potentially producing a short-term atmospheric flux of high DMS, although direct evidence for this pathway is limited^26^. DMS concentrations in Antarctic sea-ice leads have also been found to be an order of magnitude higher than in the underlying water column^27,28^.

The Western Antarctic Peninsula (WAP) is a climatically very sensitive region. During the second half of the twentieth century, a strong atmospheric warming trend was present, with a marked concurrent retreat in sea ice and warming of the upper ocean^29–32^. Over the same era, precipitation increased and winds strengthened and veered to be more northerly^33^. These changes are known not to be monotonic, but to have significant interannual variability superposed^34^. Whereas the retreat in sea ice will directly impact on the release of DMSP and DMSP-producing algae, changes in the physical environment can also impact indirectly on phytoplankton productivity and composition through changes in light and nutrient availability^32,35^.

**Figure 1:**
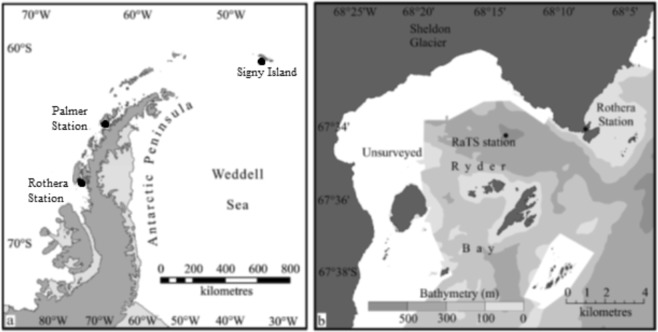
(**a**) Location of Rothera Research Station on the West Antarctic Peninsula, and (**b**) location of the Rothera Time Series (RaTS) sampling location.

Developing a full understanding of the relevant processes that impact on DMSP production requires datasets that span the pertinent timescales. So far, data from the WAP on surface water DMS concentrations are extremely limited both spatially and temporally^21,36–38^, exceptions being two extensive datasets from the Palmer Long-Term Ecological Research (LTER) program, which show increasing DMS concentrations in December, reaching relatively stable concentrations in January between 5 and 15 nmol L^−1^ and occasional maxima exceeding 25 nmol L^−1 ^^39,40^. To address this knowledge gap, we undertook a five-year study at the WAP that involved measuring water column DMS(P/O) concentrations to identify temporal variability over multiple consecutive years in both winter and summer and to associate these changes with trends in phytoplankton abundance and community composition^41^. This study was carried out at Rothera Research Station (Fig. 1), as part of the year-round observational programme for water column measurements and sea-ice observations conducted by the British Antarctic Survey (BAS) since 1998. Here, we present the DMS data, and calculate fluxes from surface-water DMS concentrations, wind speed and oceanographic data. The results are analysed in the context of existing DMS climatologies of the area. We find significantly higher fluxes than previously reported, and postulate that these high numbers are more representative of the marginal ice zone than previous quantifications.

## Results

### Oceanographic conditions

**Figure 2:**
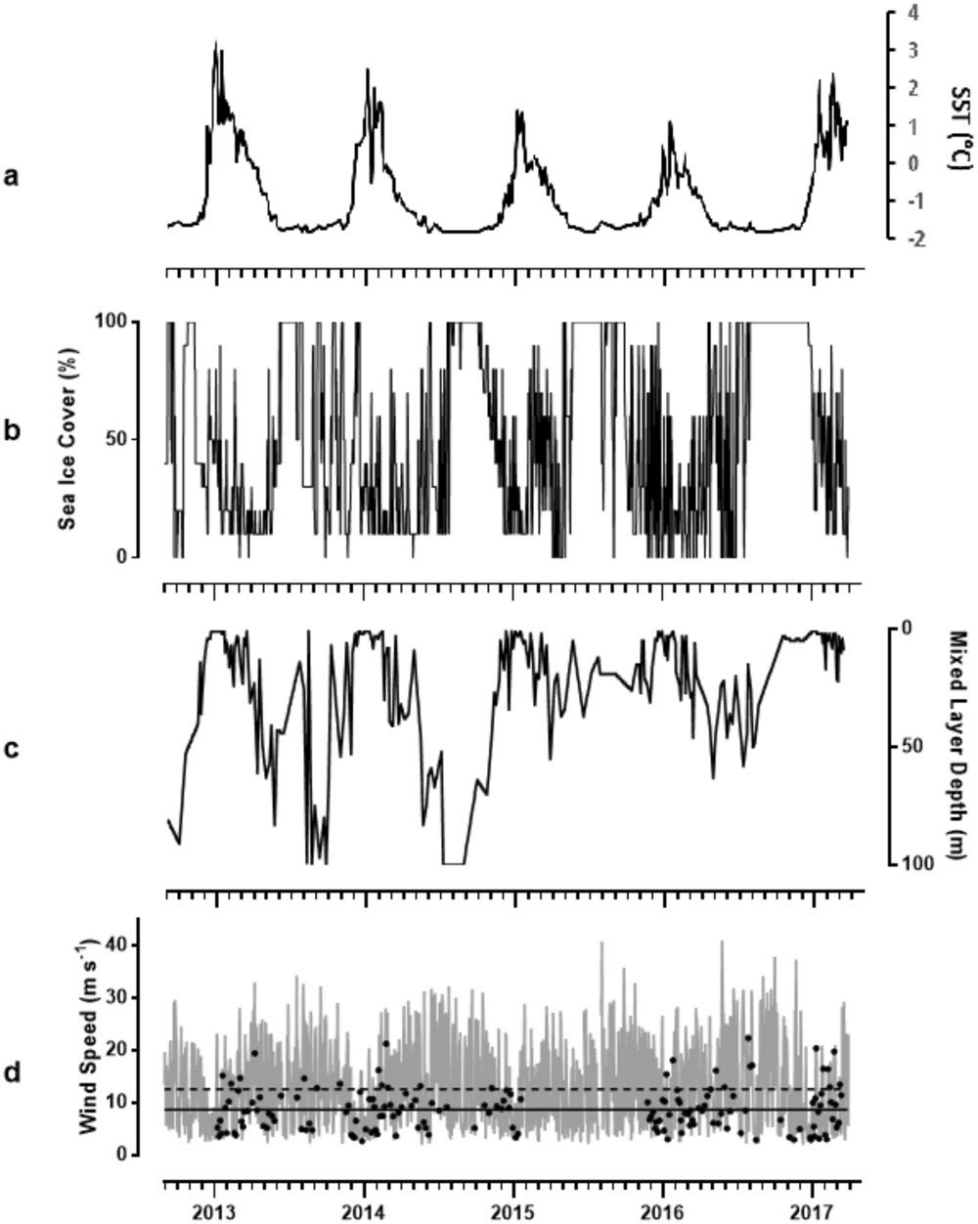
Oceanographic conditions at the Rothera Time Series (RaTS) station in Ryder Bay at the West Antarctic Peninsula during Sep 2012–Mar 2017, showing (**a**) sea surface temperature (SST), (**b**) sea-ice cover given as a percentage coverage of Ryder Bay, (**c**) mixed layer depth (MLD) in the surface 100 m and **d**) mean daily wind speed (grey), mean daily wind speed on sampling days (black points), mean 5-year wind speed (dashed line) and mean sampling-day wind speed (solid line).

Marked seasonality in sea surface temperature (SST) in Ryder Bay was identified over the five-year time-series ranging between minimum winter temperatures of −1.8 °C and a maximum summer temperature of 3.3 °C in 2012/13 (Fig. 2a), decreasing with each progressive year (2.5 °C in 2014, 1.3 °C in 2015 and 1.1 °C in 2016), followed by a markedly higher maximum summer SST in 2016/17 of 2.4 °C. The greatest variation in SST occurred during summer (November – March); temperatures above 0 °C were measured for the first time each year in mid to late December and declined to below 0 °C in late March/ April each year. The duration of 100% sea ice cover increased over the five years of our survey commensurate with the decreasing summer SST, from 86 days in 2012 to 163 days in 2016 (Fig. 2b). Despite summer 2016/17 showing an increase in SST after the decreasing trend of the previous 4 years, ice cover was the highest with an average 62% ice cover, compared to approximately 35% ice cover in all previous summers. Ice type within the bay varied throughout the seasons, as did the extent of the coverage: periods of over 80% sea ice cover were comprised of fast ice with cracks and limited open water; breakup of fast ice to below 50% coverage, and ice movement due to wind and current, resulted in a mix of brash ice of both sea ice and glacial origin and icebergs.

Over the five years, we observe a pattern of decreasing vertical mixing in the upper ocean in winter, in line with the increasing coverage and duration of sea ice. In all summers, the mixed layer depth (MLD) remained relatively shallow above 20 m (Fig. 2c). Wind at Rothera was highly variable within the range 2.2–40.7 m s^−1^ (Fig. 1d), with evidence of seasonality through stronger winds in winter. South-easterly winds were dominant, and through the five years there was no evidence of significant interannual differences. The wind speed on sampling days was identified as being on average 3.9 m s^−1^ lower than the average over the entire 5 years, as identified in Fig. 2d.

### DMS Concentrations

**Figure 3:**
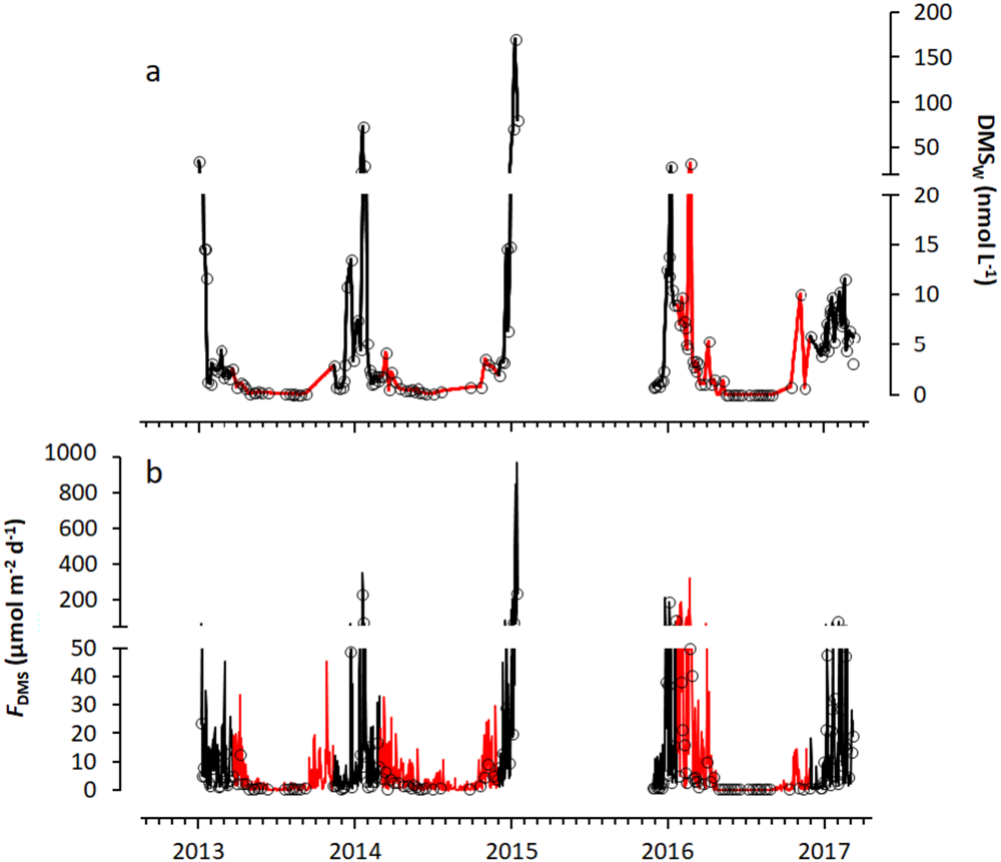
(**a**) DMS (nmol L^−1^) over the 5-year time-series in Ryder Bay at the West Antarctic Peninsula, presented as the mean over the surface 15 m and b) DMS flux (µmol m^−2^ d^−1^), calculated from DMS concentration, daily average wind speed and SST. Black solid lines represent summer (Nov–Mar) interpolated values calculated from *in-situ* measured DMS and red solid lines represent winter (Apr–Oct) interpolated values based on stored DMSPd samples analysed the following summer. Black circles represent the data points on which the interpolation was performed.

Concentration of DMS_W_ in surface waters of Ryder Bay was found to be extremely high, with concentrations exceeding 30 nmol L^−1^ in four out of five summer seasons and peaking at over 170 nmol L^−1^ in January 2015 (Fig. 3a). Strong seasonality in DMS_W_ was apparent, in addition to significant interannual variability. Peak DMS concentrations occurred mid to late January each year, except for 2017 where the maximum DMS_W_ peak was identified during February with the lowest summertime DMS_W_ maximum of 11.6 nmol L^−1^. There was no apparent relationship between DMS_W_ concentrations and the trends identified in summer SST, sea ice cover and MLD.

Wintertime (April to October) concentrations were distinctly lower than during summer, ranging between 0.1–7.1 nmol L^−1^ and averaging 0.5 nmol L^−1^ in 2013, 0.6 nmol L^−1^ in 2014 and 0.7 nmol L^−1^ in 2016.

### DMS Flux

With a 5-year averaged wind speed of 12.6 m s^−1^ (Fig. 2d), Ryder Bay is representative of areas with globally the highest wind speeds^42^. Calculating DMS transfer velocities at the sea-air interface are dependent on the gas-transfer coefficient and concentration differences at the interface (Eq. 1 in Methods). Determining the gas-transfer coefficient depends on the wind speed, but appears not to follow a simple relationship; especially at high wind speed suppression of the DMS flux is sometimes observed^43^. It was suggested that long waves suppress the water-side turbulence, thereby decreasing gas transfer of the relative soluble gases, such as DMS, whereas wave breaking and bubble-mediated exchange is of less importance for DMS exchange^43^. Given the fact that Ryder Bay is a relatively sheltered bay (Fig. 1) with limited fetch area, waves are modest. Therefore, the gas transfer versus wind relationship as determined by Nightingale *et al*. (2000) was used with the current RaTS data.

DMS flux showed the same strong seasonality as the DMS_W_ concentrations (Fig. 3b; Table 1), with summer fluxes often several orders of magnitude higher than those in winter. Over the five-year time-series, DMS flux was above the nucleation threshold^44^ of 2.5 µmol m^−2^ d^−1^ for 63% of the time. The highest mean monthly fluxes occurred during January and February (43% and 20% of total annual DMS flux respectively; Table 1). The lowest monthly mean flux occurred in August (0.4% of annual total); the month with the highest mean ice cover (Table 1).

**Table 1:** Mean monthly DMS_w_ concentrations (nmol L^−1^; averaged over the top 15 m), sea surface temperature (SST; °C) and wind speed (m s^−1^), used to determine the total monthly DMS flux from Ryder Bay (µmol m^−2^).

Month	Total No. Sampling days	Mean Daily DMS_w_ (nmol L^−1^)	Mean Daily DMS Flux (µmol m^−2^ d^−1^)	Total Monthly DMS Flux (µmol m^−2^ month^−1^)	Mean Monthly Wind Speed (m s^−1^)	Wind Speed Range (m s^−1^)	Mean Monthly Ice Cover (%)	Mean SST (°C)
Jan	33	23.6 ± 35.3	51.2 ± 125.5	1588	9.3 ± 5.6	3.0–28.6	31 ± 11	0.99 ± 0.98
Feb	33	6.0 ± 5.8	26.3 ± 45.0	736	11.6 ± 6.3	2.7–28.1	28 ± 11	0.54 ± 0.79
Mar	20	2.6 ± 1.5	10.9 ± 9.7	338	13.3 ± 6.9	2.7–29.0	30 ± 12	−0.20 ± 0.65
Apr	11	1.2 ± 1.1	7.2 ± 9.8	217	14.7 ± 6.7	3.0–32.7	25 ± 13	−1.05 ± 0.51
May	13	0.4 ± 0.3	2.0 ± 2.8	61	13.0 ± 7.9	2.2–40.7	41 ± 18	−1.55 ± 0.25
Jun	8	0.1 ± 0.1	0.6 ± 1.0	18	13.6 ± 7.9	2.4–31.6	62 ± 33	−1.71 ± 0.09
Jul	6	0.1 ± 0.1	0.9 ± 1.9	29	14.2 ± 8.2	2.4–34.0	77 ± 24	−1.73 ± 0.11
Aug	10	0.2 ± 0.2	0.5 ± 0.8	15	13.9 ± 8.7	2.5–40.5	82 ± 25	−1.79 ± 0.05
Sep	2	0.5 ± 0.3	1.5 ± 3.4	46	15.1 ± 8.3	3.5–35.5	85 ± 21	−1.73 ± 0.05
Oct	2	1.7 ± 1.4	4.5 ± 6.2	140	15.2 ± 7.6	3.1–37.6	69 ± 30	−1.61 ± 0.12
Nov	8	3.2 ± 2.2	2.9 ± 6.3	176	12.3 ± 6.7	2.5–37.1	58 ± 29	−1.52 ± 0.21
Dec	21	5.1 ± 3.8	9.6 ± 17.6	296	8.7 ± 5.4	27.4–2.2	57 ± 29	−0.20 ± 0.77
TOTAL	167			3662				

Values given are ± standard deviation. Also given is the mean monthly ice cover, given as a percentage.

During the second, third and fourth years, at least one significant spike in DMS flux was identified each year, where flux exceeded 100 µmol m^−2^ d^−1^. These spikes were related to high DMS_W_ concentrations and were important contributors to the flux totals, as each occurrence accounted for more than 20% of the mean monthly flux for the month of January. Indeed, a flux of 968 µmol m^−2^ d^−1^ was identified in January 2015 due to a combination of a mean daily wind speed greater than 20 m s^−1^ and the summer DMS peak where concentrations exceeded 80 nmol L^−1^. Despite releasing 31 mg m^−2^ sulfur to the atmosphere from Ryder Bay in a single day, this high flux still only contributed 25% of the total January flux in 2015, due to multiple consecutive days of exceptionally high flux. Summer 2016/17 did not produce a high spike in DMS as the previous summer seasons, which corresponded to lower DMS_W_ concentrations. The highest flux within this year occurred later in the season in February (77 µmol m^−2^ d^−1^ and at 16 m s^−1^ wind speed). These data were used to generate a year-round climatology of both surface DMS_W_ and DMS flux from Ryder Bay and the wider WAP region. It is estimated that the flux of sulfur in the form of DMS from Ryder Bay was 9.0 Mg S yr^−1^ (Table 2), with 89% (mean 22.7 ± 67.6 µmol m^−2^ d^−1^) produced during summer and 11% (mean 2.5 ± 5.3 µmol m^−2^ d^−1^) during the winter months.

**Table 2:** Summary of Antarctic DMS_W_ concentrations and associated fluxes (range or mean ± s.d.) in different areas of the Antarctic sea-ice zone as identified in the literature.

Location	Time of Year	Wind Speed Range (m s^−1^)	DMS_W_ Concentration (nmol L^−1^)	DMS Flux (µmol m^−2^ d^−1^)	Calculated Total Annual Sulfur Flux (Tg S yr^−1^) (area given in brackets)
Ryder Bay (This Study)	Year-round	2.2–40.7	0.05–170	0.01–968	9.02 × 10^−6^ (77 km^2^)
West Antarctic Peninsula Sea Ice Zone (This Study)	Year-round				0.06 (5.0 × 10^5^ km^2^)
Austral Polar Province (APLR) Zone (This Study)	Year-round				1.08 (9.2 × 10^6^ km^2^)
Antarctic Sea Ice Zone (This Study)	Year-round				2.18 (1.86 × 10^7^ km^2^)
Southern Ocean 60–70°S^6^	Sep-Apr (1972–2010)		0–50		0.9 (1.88 × 10^7^ km^2^)
Weddell Sea Leads^27^	Dec-Jan 2004	3.7–9.2	0.6–45.9	0.2–5.3	
Eastern Antarctic Sea-Ice Zone^72^	Dec 1998	2.0–25.0	3–31	1–101	
Eastern Antarctic Sea Ice Zone^23^	Sep-Mar (1991–1995)	11.7	7.9	49	2.7
Palmer Station, WAP^36^	Jan-Feb 1994		0.7–3.7	0.03–19.2	
Australian Antarctic Divergence 63–68°S^60,73^	Nov 1988-Jan 1989	2.5–16.0	5.3–18.8	1.0 ± 1.6	
Weddell Sea^61^	Nov-Dec 1990		2.3 ± 1.6	0.17 ± 0.09	
Weddell Sea (ISPOL)^19^	Dec 2004		0.3–1.3	14.2	
Ross Sea^74^	Nov-Dec	1.0–22.0	0.6–14.2	0.2–24.3	
Ross Sea^17^	Dec 2004 – Jan 2005, Nov 2005		4.3–65.3	0.6–9.8	
Southern Ocean^75^	Mar-Apr 2008	1.0–18.0	1.6 ± 0.7	2.9 ± 2.1	
Palmer Station, WAP^40^	Oct 2012- Mar 2013		0–20	0.3–9.3	

## Discussion

The DMS_W_ and subsequent DMS flux presented here from five years of sampling in Ryder Bay allow us to quantify the interseasonal and interannual variation in DMS emission to the atmosphere from a highly productive region, and evaluate the changes identified with variations in long-term climate trends and shorter-term climate variability, such as the El Niño event that occurred in 2016/17. The results motivate a re-evaluation of existing DMS flux models in the WAP region and have comparable implications for other regions of the Marginal Ice Zone.

In winter and early spring, DMS_W_ concentrations were generally low in water under the sea ice, due to limited *in-situ* production from water-column phytoplankton. In winter, mixed layers at the WAP are typically deep (50–150 m) due to buoyancy loss through cooling, brine rejection from ice formation, and mechanical mixing due to wind stress^45,46^. These conditions and the low-light levels in winter are unfavourable for phytoplankton growth. During periods of fast-ice cover, water temperature below −1.8 °C and low light penetration, algal communities instead thrive in close association with sea ice, living in the brine channels, porous ice layers and along the ice-sea interface^25,47^. Starting in early spring, sea ice retreats southward along the WAP as insolation and temperatures increase. Sea-ice melt together with runoff from land-based glaciers release low salinity water at the surface, resulting in stratification of the upper water column and development of a shallow (<20 m) mixed layer, despite the exposure of the sea surface to wind-driven mixing^48,49^. During mid-to-late December, a steady increase of DMS_W_ concentrations developed in all years except in 2016, when ice cover was still 100%.

**Figure 4:**
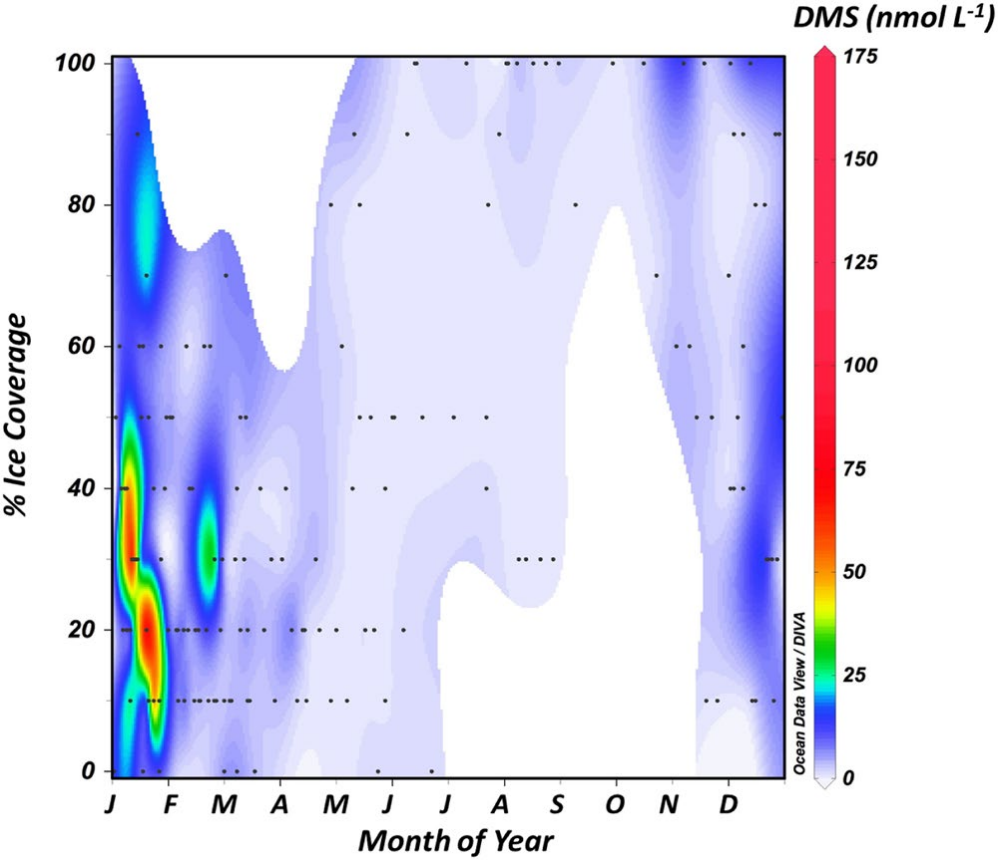
Pooled DMS_W_ concentrations (nmol L^−1^) over the 5-year time-series as a function of ice coverage (%) and time of year.

During the highest peak of DMS_W_ observed during the five years at Ryder Bay in January 2015, Stefels *et al*.^26^ calculated similar concentrations as those presented here throughout the wider Marguerite Bay area and further onto the continental shelf and also identified a strong sea-ice influence on surface water DMS(P) concentrations. Occurring in four out of the five years of DMS_W_ analysis, this January peak in DMS_W_ is judged a key part of the annual DMS_W_ cycle, covering a significant percentage of the annual DMS_W_ production in the region. In general, the highest DMS_W_ concentrations were associated with the summer break-up of sea ice, with the majority of high concentrations observed when the area coverage of ice was estimated between 10 and 40% (Fig. 4). The relatively high DMS_W_ concentration in February (Fig. 4) appeared to be an anomaly that may have been triggered by the December 2015 El Niňo. Similar coverage data during other months of the year did not resolve such high DMS_W_ concentrations, indicating that factors other than ice coverage play a role. Simo and Dachs (2002) identified a significant global relationship between DMS_W_ and Chlorophyll *a*^50^. Chlorophyll *a* is determined by fluorimetry as part of the RaTS program, and is used as a proxy for water column primary production in Ryder Bay. No relationship between Chlorophyll *a* and DMS_W_ could be identified during the five years studied, nor was there a relationship with MLD-normalised chlorophyll *a* concentration, as also proposed by Simo & Dachs (2002; Supplementary material). Further investigation into the relationship between the phytoplankton community structure, other biogeochemical parameters and subsequent DMSP, DMS and DMSO concentrations are currently undertaken^41,51^.

**Figure 5:**
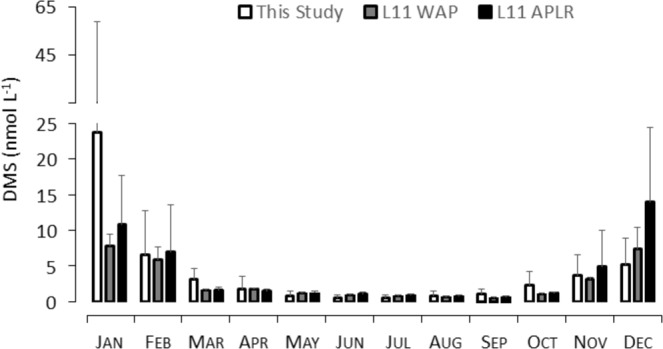
Monthly mean DMS_W_ (nmol L^−1^) for this study (open bars) as presented in Table 1, compared with the Lana *et al*.^6^ sea surface DMS climatology for both the WAP area (grey filled bars) and the entire Longhurst Austral Polar Province (APLR; solid bars), with error bars displaying the standard deviation.

To examine the DMS_W_ concentrations from Ryder Bay in a wider context, monthly mean DMS_W_ concentrations (Table 1) were compared to data from the Lana 2011 (L11) climatology of the WAP area and the Longhurst Austral Polar Province (APLR), which covers all data south of 59°S^6,52^. Strong seasonal differences were observed over both the WAP and the APLR, with maximum DMS concentrations in December (Fig. 5). Overall, within the L11 climatology, DMS_W_ concentrations for the WAP are often lower than for the entire APLR, suggesting it is an area of low DMS production. However, mean January DMS_W_ concentrations in Ryder Bay were three times higher (23.6 nmol L^−1^) than the L11-WAP data (7.8 nmol L^−1^), which resulted in annual mean surface DMS_W_ concentrations in Ryder Bay higher than the WAP (3.7 nmol L^−1^ and 2.8 nmol L^−1^ respectively). When comparing the standard deviations of the data, our data reveal a much higher variation with on average a 43% variation over the year, compared to a 6% variation for the L11-WAP data. This suggests that the L11 climatology is not accurately predicting WAP summer DMS_W_ production, and in particular is missing peak-DMS production events. This motivates improved data coverage along the WAP and further development of long-term DMS monitoring programs in order to further determine the causal factors that drive such peak events.

Sea ice cover was assumed to completely block DMS flux like a ‘cap’ over the water surface^6^. As no attempt was made to quantify DMS flux from sea ice or slush snow layers on top of sea ice, quantification of DMS flux in this study will underestimate the total Antarctic DMS-derived sulfur flux. Gas transfer from the water column can occur through partial ice cover, particularly in coastal environments such as Ryder Bay where movement of ice between the bay and the greater Marguerite Bay area results from wind, water and tidal action. Development of summer shallow MLD upon ice breakup initially suggests limited exchange to the atmosphere of DMS_W_ produced below the immediate surface layers (Fig. 2c), however spatially and temporally localized mixing processes such as ice movements, wave action, bubble entrainment and internal waves will support DMS release from deeper waters^53–55^.

Interseasonal and interannual variations in the flux of DMS were strongly pronounced but not always related to the DMS_W_ concentrations. Whereas spikes of high flux corresponded closely to the peaks in surface DMS_W_ concentration, the additional drivers – SST, wind speed and fraction of open water – resulted in a poor relationship between DMS_W_ concentration and flux over the entire five-year data set (linear regression, r^2^ = 0.371, p < 0.01). More specifically, the short-term high-flux peaks in January of each year resulted in flux rates of DMS that exceeded DMS_W_ production, and resulted in the rapid decrease in DMS_W_ concentrations the following day. Interannual variation is especially visible when comparing the 2016–2017 period with previous years, and it is suggested that connections between interannual modes of climate variability (including the El Niño/Southern Oscillation (ENSO) phenomenon and the Southern Annular Mode (SAM)) and the DMS concentrations and flux are present. Firstly, the El Niño event in 2015/2016 coincided with a period of sustained positive SAM and relatively prolonged period of medium-high DMS concentrations during summer. Secondly, an anomalously long period of fast-ice cover in Ryder Bay the following winter, together with a strong increase of SST when ice cover broke-up (Fig. 2a,b), correlated with a lack of DMS_W_ spikes in summer 2017 (Fig. 3a). External forcings are known to be significant in driving interannual changes in ocean temperature and sea ice at the WAP, and the role of SAM and ENSO has been highlighted previously^56–59^. The observed persistence of sea ice cover in Marguerite Bay noted here (Fig. 2; see also Fig. 3d of Turner *et al*.^34^) conflicts with the rapid retreat of sea ice elsewhere around Antarctica^34^, but is a known consequence of the directionality of the wind and local topography. The higher SST in Ryder Bay during the summer of 2017 (Fig. 2a) is at least partly due to reduced winter mixing and strong freshwater injection to the upper ocean upon eventual breakup and melt of the ice. This would retain incident heat from insolation in shallower layers and increase surface temperature. This resulted in a stable water column and shallow MLD. As a result, DMS flux in 2017 was still calculated to be of the same order of magnitude as the preceding four summer seasons (Fig. 3b), despite the absence of large spikes.

The flux values from Ryder Bay exceed those presented from the area of Palmer Research further north on the WAP (~64°S) by at least one order of magnitude^36,40^, as well as from other sites around Antarctica (Table 2). Of critical importance within the annual cycle were the high spikes of flux in January, potentially related to sea-ice derived DMS production. Given the short timescales over which these spikes occurred, it is highly likely that they were detected in this study only because of the high resolution of this time series; other DMS flux determinations have been from research cruises which are limited in temporal resolution^17,23,60,61^, or from studies only measuring a single summer season during which a spike may not occur^40^. High DMS fluxes above the acknowledged nucleation threshold of 2.5 µmol m^−2^ d^−1^ were observed during 63% of the year, suggesting significant periods of CCN development^44^. Within the relatively unpolluted environment of the Antarctic, nucleation from the breakdown of DMS within the Marine Boundary Layer (MBL) will be the primary source of aerosol particles^44,62–64^. A previous study demonstrated tight coupling between MBL DMS and NSS-SO_4_^2−^, particularly during periods of intense DMS flux^63^ such as we identified, suggesting that these spikes in DMS flux will further drive significant production of NSS-SO_4_^2-^ and subsequent particle nucleation. Given that high particle concentrations from nucleation events have been shown to be an important contributor to the global atmospheric aerosol budget^64^, our data provide evidence that the Antarctic coastal zone particle production has a significant impact on global particle concentrations. Our data also indicates that pulses of DMS flux are an intricate feature of Antarctic coastal areas, which can easily be missed when the frequency of data collection is insufficiently high.

In calculating DMS flux from Ryder Bay, we assumed the flux represents well a large proportion of the Antarctic coastal zone, and, in particular, that of the Marguerite Bay area, as has been demonstrated previously^26^. Therefore, we have used the interannual and interseasonal assessment of DMS flux to calculate the total atmospheric sulfur input from the coastal areas of the WAP and wider Antarctic marginal ice zone (Table 2).

Given the area and scale of the Antarctic coastal sea-ice zone and APLR, temporal and spatial availability of data on DMS_W_ concentrations and flux are low, with high variation between available measurements. In the L11 climatology^6^, surface DMS_W_ concentrations were only available for 8 months of the year, and therefore include no winter data. Using the average annual flux from the five-year time series, 1.1 million tonnes of S are released to the atmosphere from the APLR (9.2 × 10^6^ km^2^) compared to 0.9 million tonnes from the 60–70 °S latitude band (1.9 × 10^7^ km^2^) as calculated by L11^6^. Normalising for area, a twofold higher sulfur flux was calculated as our annual mean flux compared to L11, showing that the latter is significantly underestimating the Antarctic contribution to atmospheric NSS-SO_4_^2−^ input. In addition, we highlight the south-western side of the WAP as a significant source of sulfur.

Jarníková and Tortell (2016)^14^ utilised a larger DMS_W_ dataset in the Southern Ocean, including a better parameterisation of DMS_W_ and region-specific parameters, to generate a new Southern Ocean DMS summer climatology (J16). During December to February, when our mean DMS flux from Ryder Bay was 30 µmol m^−2^ d^−1^, J16 calculated western WAP DMS flux was below 10 µmol m^−2^ d^−1 ^^14^. It has been previously discussed^26^ that the basin-scale methodology used in the J16 climatology does not account for large and dynamic fluctuations in the DMS_W_ concentration and DMS flux shown in the Ryder Bay time series. It is apparent that the important pulses of DMS, leading to potential nucleation events and high particle production, are missed if relatively small data sets are averaged over longer time periods. We show that the data points capped in climatologies should be considered in the model calculations, as these are recurring features of the Antarctic coastal zone. In a global atmospheric circulation model, based on the existing DMS climatologies, it was established that despite variation in spatial and temporal production of DMS, the global mean radiative effect of sulfate is linearly proportional to the global mean surface flux of DMS^44,65^. It remains an open question what the radiative effect of the high spikes of DMS and subsequent flux as found in this study will be if incorporated into climate models.

## Conclusion

We present here the DMS_W_ concentrations from the longest running time-series studying organic sulfur cycling in the world, and demonstrate the first ever evaluation of interseasonal and interannual variability in DMS_W_ and associated flux from Antarctica. *In situ* concentrations of DMS_W_ and derived DMS flux were compared to those extracted for the WAP from existing DMS climatologies, and were significantly higher than models predict during January, the most productive time of the annual cycle. Our data also indicate that DMS flux is above the nucleation threshold of 2.5 µmol m^−2^ d^−1^ for >60% of the time. Together with extremely high spikes of DMS flux during the Austral summer, this would result in significant production of NSS-SO_4_^2−^ and subsequent particle formation. December was the month of maximum DMS concentrations in the L11-climatology (Fig. 5), whereas our Ryder-Bay data showed a persistent January maximum associated with the marginal ice zone. The L11-December maximum was driven by a very strong DMS signal along the north and east of the WAP and around the South Orkney Islands^6^. Given the seasonal retreat of sea ice southward along the Peninsula, it can be expected that springtime sea ice-derived DMS production will commence earlier in more northerly latitudes of the APLR compared to the more southerly Peninsula, and spread south, reaching Ryder Bay in January for peak DMS_W_ concentrations.

Variations in winter sea ice coverage, SST and MLD within Ryder Bay were drivers of spring and summer surface water conditions, following the patterns previously identified by Venables and Meredith (2014)^66^. Of particular interest during this study was the significant shift in ENSO and SAM modes of climate variability in late 2015, which resulted in changes in SST and ice formation around the WAP during winter 2016 and further into the following summer. External forcings such as these have previously been significant in driving interannual changes in SST and sea ice^56,57,59^, and we show that the resulting changes have the potential to effect production of DMS_W_. Further long-term measurements and analysis of both DMS_W_ and DMS flux are essential to improve mechanistic understanding of the changes within the sulfur cycle during long-term climate variability and to understand the driving forces of DMS spikes that drive an important part of the total flux.

## Methods

### Sample collection and analysis

Samples for DMS and physical parameters were collected 2–3 times per week throughout five summer seasons (January 2013 through to March 2017) from the primary Rothera Time Series (RaTS) site at 67.570°S 68.225°W in south-facing Ryder Bay (Fig. 1, 520 m depth), approximately 4 km to the west of Rothera Research Station. During the winter seasons of 2013, 2014 and 2016, samples were taken and stored for analysis in the following summer. In general, during summer months, samples were taken from the surface, 5 m and 15 m, however during the 2014–15 summer season and adjacent winters, no surface samples were taken. When algal biomass was very low in winter, only 15m-depth samples were taken.

The surface-water sample was taken from a small boat using an 80 mL plastic syringe with teflon tubing from approximately 5 cm depth; deeper samples were collected using an 8 L Niskin bottle and hand-winch. In case of 100% sea-ice coverage, samples were collected through an ice-hole from a sledge-mounted winch. Two samples from each depth were collected in 70 mL amber glass vials, using silicone tubing from the outlet of the Niskin bottle; vials were filled from the bottom and overflowed for at least twice the volume of the vial before the tubing was carefully removed. Vials were sealed with Teflon-lined screw caps, with care taken to avoid bubble formation, and were protected from light and temperature changes in a cooler filled with surface water.

Alongside discrete water sampling, continuous data collection for physico-chemical properties of the RaTS sampling site was undertaken. A Seabird 19+ conductivity, temperature and depth (CTD) instrument, combined with a WetLabs in-line fluorometer for Chlorophyll *a* fluorescence and LiCOR photosynthetically active radiation (PAR) sensor, was deployed in a single 500 m cast. Data was collected on the down-cast of the instrument, ensuring the shadow of the boat was not interfering with the irradiance data. The surface 1 m measurements were used to determine SST and salinity for flux calculations. The mixed layer depth (MLD) was calculated as the depth at which the density difference relative to the surface is 0.05 kg m^−3^, consistent with previous RaTS studies^48^. Full details of RaTS sampling is given in Clarke *et al*.^49^. Wind speed at Rothera was obtained from meteorological sensors on station^67^, and averaged over 24 hours. Sea ice cover in the bay was evaluated visually on a daily basis by observers on station: 100% sea ice cover was characterised by zero visible open water, and zero percent was no visible ice. Sea Ice trends have previously shown good agreement with wider-scale satellite-derived methods^46,48^ Fractional ice cover in between the two extremes was comprised of occasional large ice sheets, smaller floes, ice bergs and brash ice. The ice cover would move throughout the bay during the day with wind and water movements.

Samples were delivered to the laboratory at Rothera within two hours of collection and were stored at 2 °C in the dark. During summer, 50 µL of a known solution of ^13^C-DMS was added to each amber sample vial, in order to quantify DMS loss during further processing: 8 mL was taken from the vial and stored in a teflon-stoppered vial for quantification of the total amount of ^13^C-DMS added. The remaining contents (62 mL) of the amber vial were gravity filtered through a 47 mm Whatmann glass microfiber GF/F filter under dim light conditions, with the process stopped after approximately 20 mL had filtered, prior to the filter being exposed to the atmosphere. Two 8 mL replicates were collected in 20 mL glass, Teflon-stoppered vials for immediate analysis of DMS. DMS was injected into a Proton-Transfer-Reaction Time-Of-Flight mass spectrometer (PTR-TOF-MS 8000, Ionicon Analytik GmbH, Innsbruck, Austria) by purging at a flow rate of 150 ml min^−1^ through the sample within the vial. A full description of the PTR-TOF-MS method is described elsewhere^26^. The instrument was calibrated every 10 samples using fresh ~6 µM working DMS standard made in Milli-Q from which ~300 pmol DMS was injected. DMS standards were regularly cross-referenced with deuterated D_3_-DMSP standards. The instrument has a detection limit of 1–2 pmol. Final DMS concentrations were calculated after correction for the amount of ^13^C-DMS lost during filtration.

During the Austral winter (Apr–Nov) of 2013, 2014 and 2016, water samples were collected and stored for DMS + DMSP (total and dissolved), with no direct analysis of *in-situ* DMS. Two 70 mL amber glass vials were filled from a Niskin bottle typically collected from 15 m depth, but also from 5 m depth when CTD-fluorescence measurements indicated increased algal biomass. The sample was gravity filtered in a similar way as described for the summer samples. A 10 mL subsample of the filtrate for determination of DMS plus the dissolved DMSP fraction (DMS(P)_d_) was collected in a 20 mL glass vial, a pellet of NaOH was added and the vial crimp sealed. Samples were stored at −20 °C and analysed during the following summer season. For these winter measurements, the assumption was made that the DMS + DMSP_d_ concentration was the absolute upper limit of potential winter DMS water concentrations and is therefore likely to be an overestimation of DMS. Over the course of the five summer seasons, DMS was on average 2.5 times higher in concentration than DMSP_d_, suggesting that the inclusion of DMSP_d_ in the winter values will overestimate winter DMS by a mean of 40%. During the winter of 2016, loss of DMS during storage was tested: all samples received an addition of ~200 pmol D_3_-DMSP standard, before adding the NaOH pellet. Ten individual D_3_-DMSP working standards were prepared in March 2016, analysed to provide a baseline concentration (t_0_) and stored in crimp-sealed vials. Every month during winter, a new standard vial was opened for addition to the samples and control samples prepared and stored (t_1_). Samples from all remaining standards were again prepared upon return to summer-sampling protocol in November 2016 and immediately analysed (t_end_). This analysis was compared with the t_0_ and t_1_ analyses, to quantify loss during use of the standard. All samples collected for DMS(P)d during winter were compared to the t_0_ and t_end_ standards, and the concentration of each sample adjusted for the percentage recovery of D_3_-DMS. Mean percentage recovery across the entire winter was 93 ± 14%.

Concentrations were averaged over the upper 15 m, with the non-constant depth intervals between samples accounted for in the averaging. This integrated concentration was divided by the depth, to give the mean DMS concentration and was assumed to be the concentration available at the surface for flux. This mean concentration was preferred to a direct measurement of DMS at the surface to remove spatial variability in surface values due to ice cover, and also to provide a best comparison with existing literature. The 15 m cut-off point was selected to provide the best comparison between summer and winter. DMS concentrations for non-sampling days were interpolated from existing values, assuming a linear relationship between concentrations on each sampled day.

### Sea-Air flux of DMS

The DMS flux (*F*_DMS_) to the atmosphere (µmol m^−2^ d^−1^) was calculated for each day, using Equation (1):

1 $${F}_{{\rm{DMS}}}={k}_{{\rm{DMS}}}({{\rm{DMS}}}_{{\rm{W}}}){(1-A)}^{0.4}$$

where DMS_W_ is the concentration of DMS (nmol L^−1^) and *A* is the fraction sea-ice cover. Due to the nature of sea-ice, it was expected that even during periods of extreme ice cover, flux would still be occurring through leads and brine channels^68^, and therefore the minimum open water fraction was set to 0.01^69^. The scaling of 0.4 allows for the determination of the effect of sea-ice cover on the flux, and is provided by the work of Loose *et al*. (2009)^54^. *k*DMS is the gas transfer velocity (cm^−3^ hr^−1^) as based on the model of Nightingale *et al*.^70^, according to the method given in Simó and Dachs^50^:

2 $${{\rm{k}}}_{{\rm{DMS}}}=(5.88{u}^{2}+1.49u){({\rm{Sc}})}^{-0.5}$$

where *u* is the wind speed (m s^−1^) and Sc is the Schmidt number (cm^2^ sec ^−1^), which is dependent upon sea surface temperature (t) and salinity as described by the model of Saltzman *et al*.^71^:

3 $${\rm{Sc}}=2674.0-147.12t+3.72{t}^{2}-0.038{t}^{3}$$

## Supplementary information

Supplemental

## Data Availability

The datasets generated during and/or analysed during the current study are available from the corresponding author on reasonable request.
