# Supplementary material for: Extreme spikes in DMS flux double estimates of biogenic sulfur export from the Antarctic coastal zone to the atmosphere
Source: Sci Rep. 2019 Feb 19;9:2233. doi: 10.1038/s41598-019-38714-4 (PMC6381205; doi:10.1038/s41598-019-38714-4)
Supplement: Supplementary file 1 — Supplemental [file 41598_2019_38714_MOESM1_ESM.pdf]

Extreme spikes in DMS flux double estimates of biogenic sulfur export from the Antarctic coastal zone to the atmosphere

Webb, A.L.[a,b]*, van Leeuwe, M.A.[b], den Os, D.[b,c], Meredith, M.P.[d], Venables, H.[d] Stefels, J.[b]

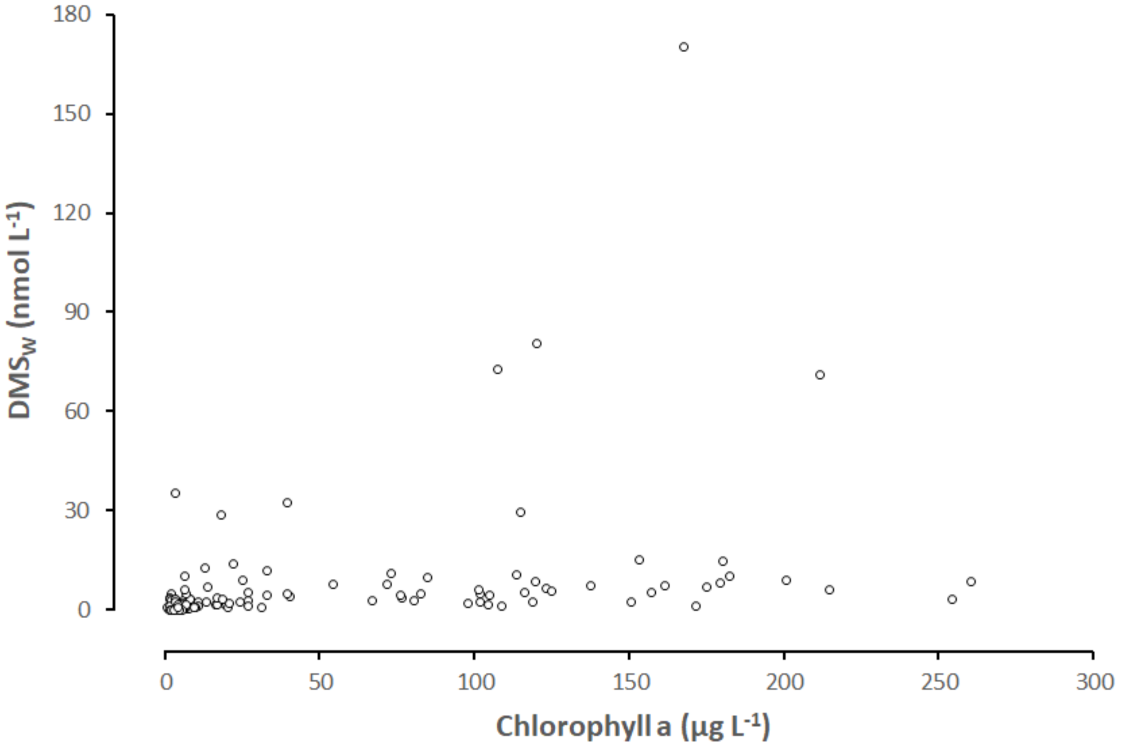

Figure S1. Scatterplot showing the relationship between Chlorophyll *a* (ug L^{-1}) and DMS_W (nmol L^{-1}). Each datapoint represents a measurement and not an interpolated value.

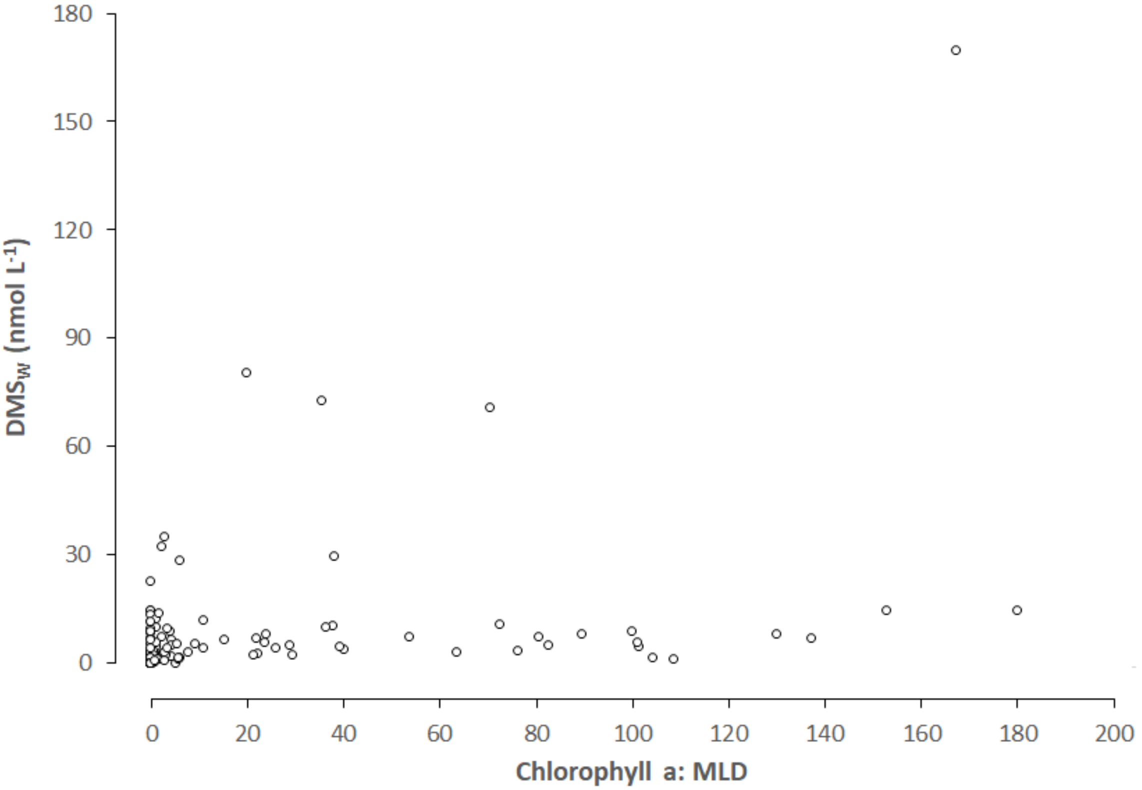

Figure S2. Scatterplot showing the relationship between the Chlorophyll a: MLD ratio and DMS_W (nmol L^{-1}). Each datapoint represents a measurement and not an interpolated value.
